# The Histone Methyltransferase Ash1l is Required for Epidermal Homeostasis in Mice

**DOI:** 10.1038/srep45401

**Published:** 2017-04-04

**Authors:** Gang Li, Zhisheng Ye, Cheng Shi, Ling Sun, Min Han, Yuan Zhuang, Tian Xu, Shimin Zhao, Xiaohui Wu

**Affiliations:** 1 State Key Laboratory of Genetic Engineering and National Center for International Research of Development and Disease, Institute of Developmental Biology and Molecular Medicine, Collaborative Innovation Center for Genetics and Development, School of Life Sciences, Fudan University, Shanghai 200433, China; 2 Howard Hughes Medical Institute, Department of Molecular, Cellular, Developmental Biology, University of Colorado, Boulder, CO 80309, USA; 3 Department of Immunology, Duke University Medical Center, Durham, NC 27710, USA; 4 Howard Hughes Medical Institute, Department of Genetics, Yale University School of Medicine, New Haven, CT 06536, USA

## Abstract

Epidermal homeostasis under normal and healing conditions are critical for the physical and functional maintenance of the skin barrier. It requires a proper balance between keratinocyte proliferation and differentiation under genetic and epigenetic regulations. Here we show that mice carrying a hypomorphic mutation of the histone methyltransferase *Ash1l* [(absent, small, or homeotic)-like (Drosophila)] develop epidermal hyperplasia and impaired epidermal stratification upon aging. In adult mutants, loss of *Ash1l* leads to more proliferative keratinocytes in disturbed differentiation stages. After wounding, *Ash1l* mutation leads to delayed re-epithlialization but increased keratinocyte proliferation at the wound edge. Elevated c-Myc expression could be observed in both aged and wounded mutant tissues. Taken together, these observations revealed an important role of the epigenetic regulator *Ash1l* in epidermal homeostasis.

The epidermis is a self-renewing, stratified squamous epithelial tissue consisting of one basal layer and several suprabasal layers of keratinocytes. The basal layer is composed of stem cells and transit amplifying cells. After derived from stem cells, transit amplifying cells undergo limited rounds of cell divisions before initiating terminal differentiation and moving outwards to form suprabasal layers with committed keratinocytes^1^,^2^. In mice, the formation of suprabasal layers begins around embryonic day 15 (E15) and a functional barrier was readily established at birth. An exquisite regulation of the balance between skin cell proliferation and differentiation is critical not only for the maintenance of epidermis, but also for skin regeneration after injury^3^. Upon injury, stem cells are activated to generate different cell types that could reepithelialize and close the wound^3^,^4^,^5^,^6^. In the absence of re-epithelializaiton, as in chronic wounds, the loss of barrier integrity provides a portal for infection or wound reoccurrence^7^.

Epigenetic mechanism has been shown to regulate epidermal homeostasis in a cooperative pattern^8^,^9^. The polycomb group (PcG) proteins, first identified in *Drosophila melanogaster* through their roles in silencing homeotic (*Hox*) genes^10^, have been revealed to play important roles in regulating epidermis development and homeostasis^9^,^11^,^12^. In basal layer stem cells, histone modification imposed by PcG proteins prohibits late differentiation genes from transcription. While in suprabasal layers, late differentiation genes lose H3K27 tri-methylation (H3K27me3) modification and become activated. Trithorax group proteins can antagonize PcG dependent gene silencing by affecting histone methylation^13^,^14^. However, their regulatory mechanism was largely unexplored in epidermis.

*Ash1l* is the mammalian homolog of *Drosophila Ash1* (absent, small, or homeotic), a well-known component of the Trithorax group proteins. *Ash1l* encodes a SET domain–containing protein with H3K36 methyltransferase activity^14^,^15^,^16^,^17^,^18^,^19^,^20^. Correspondingly, *Ash1l* has been shown to have regulatory roles on different Hox genes by antagonizing H3K27me3 in embryonic stem cells^13^. Considering the importance of epigenetic regulation in epidermis, we herein examined the role of *Ash1l* in epidermal development and wound healing in mice. We report that, wound healing in *Ash1l* mutant mice is disturbed due to delayed re-epithelialization, increased proliferation and altered expression of keratinocyte markers. The chronic wound phenotype in mutant is possibly due to elevated c-Myc expression in the wound adjacent mutant epidermis.

## Results

### Disruption of *Ash1l* leads to epidermal hyperplasia in aged mice

**Figure 1:**
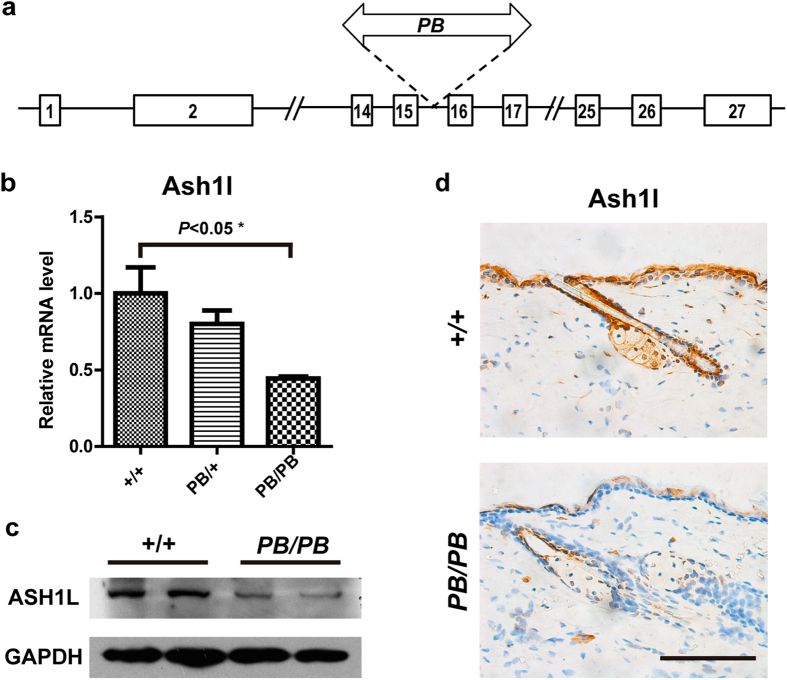
Disrupted *Ash1l* expression in *Ash1l*^*PB*/PB^ mice. (**a**) Schematic representation of the *Ash1l*^*PB*^ allele that carries a *PB[RFP]* insertion in the 15^th^ intron. Exons are labeled as numbered boxes. (**b**) Real-time RT-PCR analysis of *Ash1l* expression in the wild-type (+/+, lateral-cut bar, n = 3), heterozygous (PB/+, gridding bar, n = 3) and homozygous (PB/PB, diagonal bar, n = 3) mutant embryos. (**c**) Western blot showing decreased Ashl1 proteins in the back skin at the age of 21 days. (**d**) Immunohistochemistry staining of skin sections from 7-week-old mice revealed weaker expression of ASH1L (brown) in the granular layer and the sebaceous glands of *Ash1l*^*PB/PB*^ mutants. Brown: *Ash1l*; Blue: cell nuclei; Scale bar = 100 μm.

**Figure 2:**
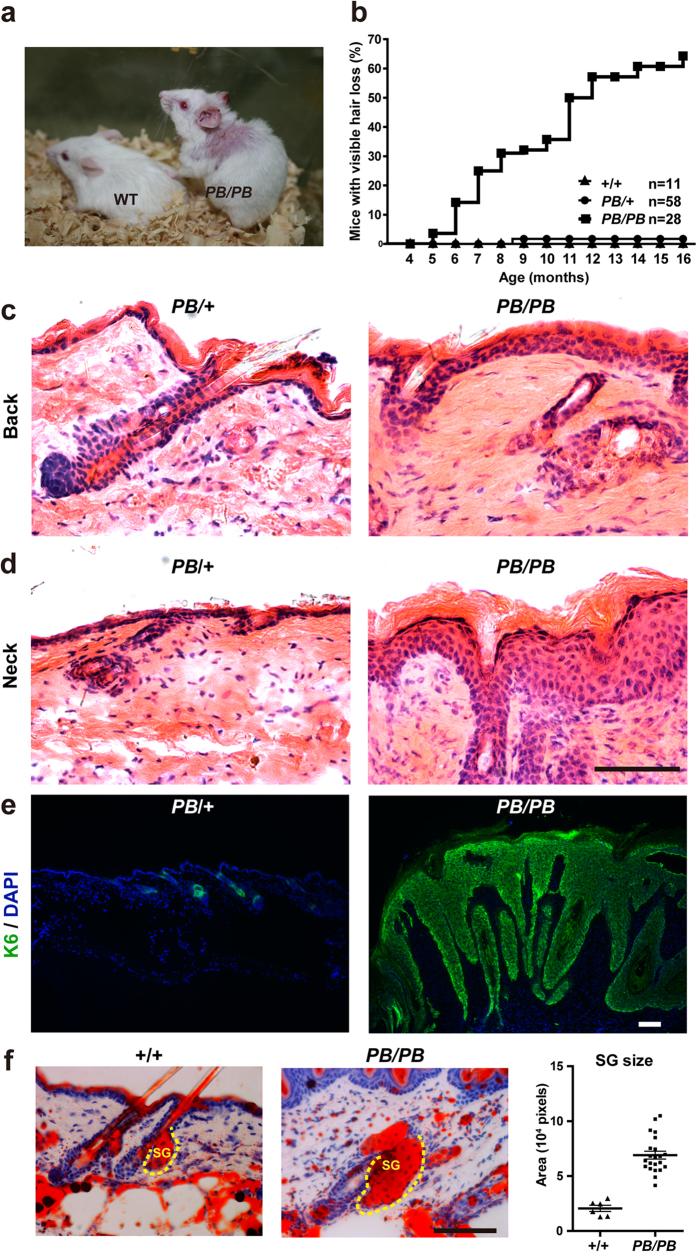
*Ash1l* mutation leads to skin hyperplasia upon aging. (**a**) Hair loss detected in a 13-month-old male *Ash1l*^*PB*/PB^ mouse, but not in its wild-type littermate. (**b**) Penetrance of hair loss increased upon aging. (**c**) Histology analysis revealed a slightly thickened epidermis in unaffected skin in 13-month-old *Ash1l*^*PB*/PB^ mice (n = 5) than in heterozygous mice of the same age (n = 3). (**d**) Epidermal hyperplasia detected in lesion area of the same group of mice in (**c**). (**e**) Immunofluorescent staining detected upregulated expression of the keratinocyte proliferation marker K6 in lesion area of 13-month-old *Ash1l*^*PB*/PB^ mice (n = 3) than in heterozygous mice of the same age (n = 3). (**f**) Oil red O staining of a significantly enlarged sebaceous gland from the lesion area of 13-month-old *Ash1l*^*PB*/PB^ mice and their wild-type littermates. Scale bar = 100 μm.

We isolated an *Ash1l* mutation from a large-scale insertional mutagenesis project with the *piggyBac (PB*) transposon. The mutant allele (*Ashl1*^*PB*^) carries a *PB* insertion in the 15th intron of *Ash1l* (Fig. 1a), resulted in reduced gene expression. Quantitative RT-PCR revealed a 60% decrease of *Ash1l* transcription in homozygous embryos (Fig. 1b). Similar changes were also observed by Western and immunohistochemistry (IHC) analysis in the epidermis of 7-week-old homozygous animals (Fig. 1c,d). *Ashl1*^*PB/PB*^ mice had normal external morphology when they were young, but gradually lost hair since four months of age (Fig. 2a,b). Skin lesions initially emerged in areas under chronic mechanical stimulations, especially in the cheek and upper back regions surrounding ears, then exacerbated and sometimes spread to the lower back in older mice. Histological analysis revealed thickened epidermis in areas with normal external morphology (Fig. 2c), which became more prominent in the hair loss region (Fig. 2d). Strong expression of the keratinocyte proliferation marker keratin-6 (K6), enlargement sebaceous glands (SGs), and abnormal hair follicles (HFs) were observed (Fig. 2e,f). The epidermal hyperplasia in *Ash1l* mutants suggested that *Ash1l* is required for epidermal homeostasis.

### *Ash1l* mutation causes disturbed keratinocyte differentiation in adult mice

Epidermal homeostasis is maintained by an exquisite balance between cell differentiation and cell proliferation. To find out which process is responsible for abnormal epidermal homeostasis in *Ash1l* mutants, we first examined cell differentiation. In wild-type mice, surface ectoderm cells commit epidermal cell fate by expressing basal keratin 14 (K14). K14 positive cells form the basal layer that contains epidermal stem cells and transient amplifying (TA) cells. Keratinocytes expressing suprabasal keratin 1 (K1) are then differentiated from the newly formed basal layer to form the spinous layer at embryonic day 15 (E15). By E17, both granular and stratum corneum layers could be observed with intensive signals of the terminal differentiation marker loricrin, which indicates the acquisition of skin barrier function^21^ (Fig. S1a,c). *Ash1l* mutants displayed similar expression pattern of K14, K1, and loricrin as those of the wild-type littermates at E15 and E17, respectively (Fig. S1a,c). Skin permeability assay also revealed a comparable cornified envelope maturation and permeability pattern between *Ash1l* mutants and wild-type animals at E16.5^22^ (Fig. S1b). These results indicated that loss of *Ash1l* did not affect cell differentiation during early skin development.

**Figure 3:**
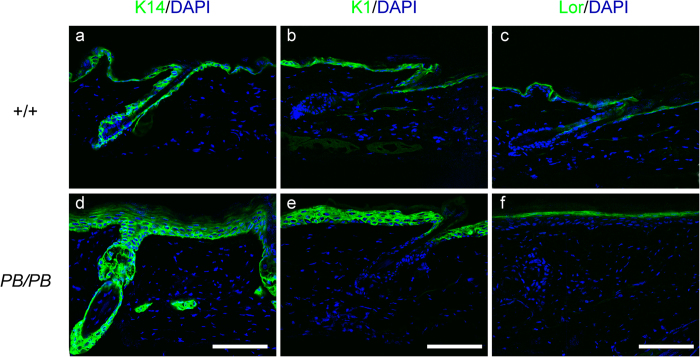
Disturbed keratinocyte differentiation in *Ash1l*^*PB*/PB^ mice. Immunofluorescent staining at the age of four months detected keratin 14 expression in both basal and suprabasal layers of the homozygous mutants (**d**), but only in the basal layer of the wild-type littermates (**a**). Compared with that in the wild-type (**b**), keratin 1 expression was also expanded in suprabasal layers of the homozygous mutants (**e**). Similar distribution of loricrin was observed in Ashl1 mutants (**f**) and wild-type littermates (**c**). Three mice were examined in each group.

In aged adult mutants, K14, K1, and loricrin were all detectable as well (Fig. 3d–f), suggesting keratinocyte differentiation could be processed to the terminal stage. However, both K14 and K1 were expressed with a more diffused pattern in the skin lesions than those in wild-type animals. K14 expression existed not only in the basal layer, but also in the suprabasal regions (Fig. 3d). Similarly, K1 expression could also be detected in multiple layers of suprabasal keratinocytes in *Ash1l*^*PB/PB*^ epidermis (Fig. 3e). Taken together, these results suggested a disturbed epidermal differentiation in adult *Ash1l* mutants.

### *Ash1l* mutation causes over proliferation of keratinocyte in adult mice

**Figure 4:**
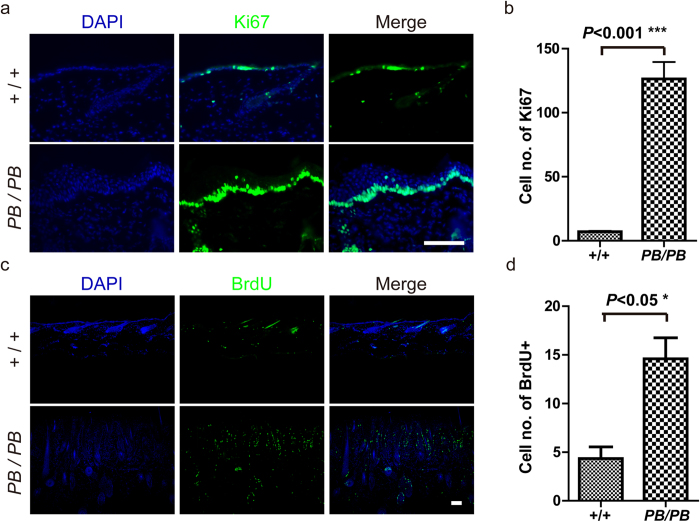
Keratinocyte overproliferation in *Ash1l*^*PB/PB*^ mice. (**a**,**b**) Immunofluorescent staining showing elevated expression of the proliferation marker Ki-67 in skin lesion of 12-week old mutants (n = 4) than in wild-type mice of the same age (n = 4). (**c**,**d**) BrdU (green) labeling detected more proliferating cells in skin lesion area of one year old mutants (n = 3) than in their wild-type littermates (n = 4). Scale bar = 200 μm.

We next examined the effects of *Ash1l* on keratinocyte proliferation. Immunostaining of the cell proliferative marker Ki67 in skin from the neck area revealed more Ki67+ cells in the basal layer of 12-week-old *Ash1l*^*PB/PB*^mice (Fig. 4a). Compared with those in wild-type littermates, more than 10 folds of Ki67+ cells could be observed in the mutants (Fig. 4b). This result indicated a marked increase of hyperproliferative keratinocytes before the onset of skin lesions in *Ash1l*^*PB/PB*^ mice. Consistently, we observed more proliferative cells in the skin lesion area of 12-month-old mutant mice. Two hours after BrdU labeling, more than four folds of BrdU positive cells could be observed in *Ash1l*^*PB/PB*^ epidermis (Fig. 4c,d). Thus, *Ashl1* mutation promotes cell proliferation in epidermis.

Overproliferation of epidermal cells could result from the recruitment of more stem cells into the proliferative cycle, or increased rounds of TA cell division^23^. Epidermal stem cells are known to have a long period between cell division, such that they are the only cells that carry BrdU labels after weeks^6^. We thus labeled epidermal cells by BrdU at postnatal day 3 (P3) and checked the number of label-retaining cells (LRCs) at the age of twelve weeks. Both *Ash1l*^*PB*/*PB*^ mice and their wild-type littermates had similar LRCs at this stage, suggesting that stem cells are less likely involved in the progression of skin lesions (Fig. S2).

### *Ash1l* ^*PB/PB*^ mice have wound healing defects

**Figure 5:**
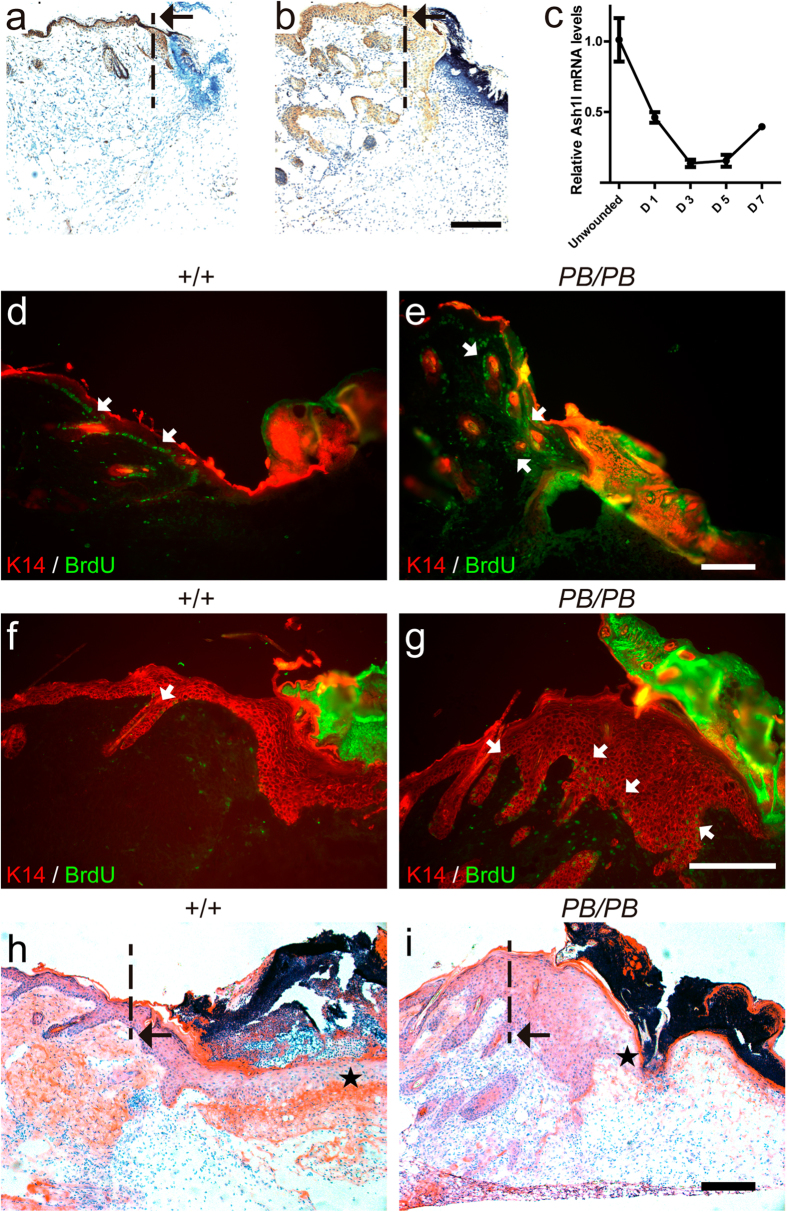
*Ash1l*^*PB/PB*^ mice exhibit impaired wound healing. (**a**) Immunohistochemistry staining of *Ash1l* (brown) around the punch biopsy one day after wounding. (**b**) Same analysis performed two days after wounding revealed a more diffused pattern of *Ash1l* expression. (**c**) Real-time RT-PCR showing dynamic *Ash1l* expression around the wound edge area. Relative expression were calculated with *Actin* as the internal control and normalized to levels before wounding. (**d**–**g**) BrdU (green) and K14 (red) staining of skin wounds in wild-type and *Ash1l*^*PB*/PB^ mice. Two days after wounding, similar amounts of proliferating cells (white arrows) between wild-type (**d**) and mutant (**e**) mice were observed around the wound edge. Four days after wounding, more proliferating cells could be observed in the mutant sample (**g**) than in wild-type mice (**f**). (**h**,**i**) Compared with wild-type littermates (**h**), *Ash1l*^*PB*/PB^ mice (**i**) accumulated more keratinocytes around the edge, but had less cells spreading inside the lesion(asterisks) four days after wounding. Four mice were sampled for each genotype at each time point. Dashed line and black arrow: original position of the wound edge. Scale bar = 50 μm.

The skin lesion in mutant mice initially developed in the neck, a place that is easily scratched by hind paws. This led us to suspect whether *Ash1l* is involved in the healing response. We utilized three-millimeter punch biopsy induced wounds to address this question. After punching, keratinocytes start proliferation to develop a re-epithelial layer under blood clots within two days. They will then form a fully re-epithelialized layer around 4 days after wounding (D4) (Fig. 5b,h)^24^. We detected a dynamic change of Ash1l expression during wound healing. Immunohistochemistry staining reveals intense Ash1l signals in keratinocytes in wound edge at D1 (Fig. 5a). Weaker Ash1l signals could be observed with a broader distribution at D2 (Fig. 5b). Real-time RT-PCR confirmed decreasing of *Ash1l* expression during the first three days of wound healing, which started to recover after D5 (Fig. 5c). We also used BrdU labeling assay to examine the proliferation of keratinocytes at the edge of wounds. At D2, comparable number of proliferating cells were detected in the basal layer between *Ash1l*^*PB/PB*^ mice and their wild-type littermates (Fig. 5d,e). However, more proliferating cells could be easily recognized in *Ash1l*^*PB*/PB^ than in wild-type animals at D4 (Fig. 5f,g). Consistent with this observation, more keratinocytes were observed at the edge of wounds in *Ash1l*^*PB/PB*^ mice. However, they were less capable of spreading into the wound to form the re-epithelial layer (Fig. 5h,i). These observations indicated that loss of *Ash1l* hamper wound healing.

### ASH1L antagonizes H3K27 tri-methylation and c-Myc expression

**Figure 6:**
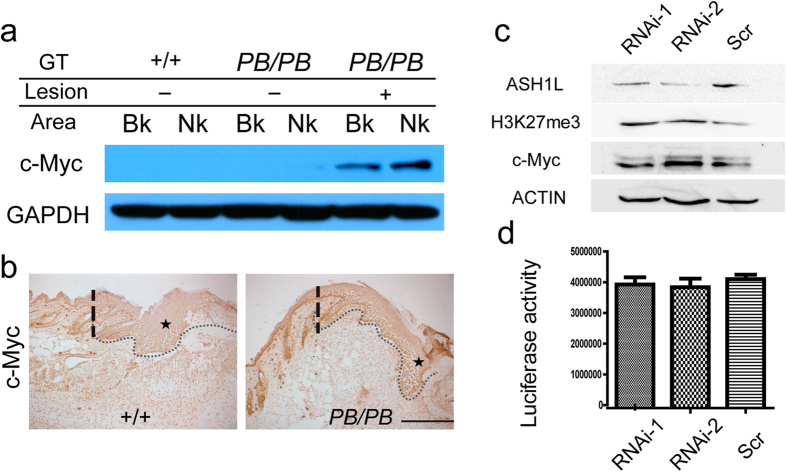
*Ash1l* affects c-Myc expression and H3K27 tri-methylation. (**a**) Western blot of skins from 13-month-old wild-type and *Ash1l* mutant mice. Nk: neck; Bk: Lower back. (**b**) Immunohistochemistry analysis of epidermal c-Myc expression (asterisks) four days after wounding. Dashed line: original position of the wound edge; dotted line: epidermal–dermal boundary. (**c**) Western analysis of 293 T cells transfected with *Ash1l* (RNAi) or scrambled (Scr) siRNA. Knockdown of *Ash1l* increased global H3K27 tri-methylation (H3K27me3) and c-Myc expression. (**d**) *Ash1l* knockdown did not alter the activity of luciferase driven by a *c-Myc* promoter (n = 3).

The abnormalities found in *Ash1l*^*PB/PB*^ mutants were reminiscent of the previous findings in c-*myc* transgenic mice^6^,^25^,^26^. Both animals showed increased cell proliferation but disturbed cell differentiation in epidermis. Upon wounding, both mutants displayed keratinocyte overproliferation but delayed re-epithelialization^6^. To explore the functional relation between *Ash1l* and *c-Myc* in skin homeostasis, we first examined c-Myc expression in the epidermis of *Ash1l*^*PB*/PB^ mice. Western blot showed that c-Myc was upregulated in hyperplasia epidermis, but not in non-hyperplasia *Ash1l*^PB/PB^ or wild-type epidermis (Fig. 6a). Immunohistochemistry staining revealed higher c-Myc expression in post-wounding area as well (Fig. 6b).

As a methyltransferase, *Ash1l* is able to methylate histone H3K36^18^,^27^, which has been shown in stem cells to be able to antagonize H3K27 tri-methylation, an reaction that is implemented by Polycomb group (PcG) proteins^14^,^27^,^28^. On the other hand, H3K27 tri-methylation is known to be a critical step for PcG dependent activation of *c-Myc* in tumor cells^29^. We found that knock down of *Ash1l* in 293 T cells increased global H3K27 tri-methylation and c-Myc expression (Fig. 6c). At the same time, direct transcription regulatory effect of *Ash1l* on the *c-Myc* promoter was not observed by the luciferase assay (Fig. 6d). Taken together, it is likely that *Ash1l* stimulates c-Myc expression in epidermis by altering histone modifications.

## Discussion

In this study, we have shown that disruption of *Ash1l* leads to disturbed epidermal differentiation, excessive keratinocyte proliferation, defective wound healing, and skin hyperplasia in adult mice. These results suggest *Ash1l* as an important regulator of epidermal homeostasis, which is consistent with previous reports of the essential roles of epigenetic regulators in epidermal cell renewal and differentiation. The PcG proteins are known to maintain proliferative potentials in progenitor cells. For example, EzH2 and Bmi-1, core elements of the PcG repressive complexes, could maintain the proliferative status epidermal stem cells by introducing repressive H3K27m3^11^,^12^, while ablation of major PcG proteins led to defective proliferation and premature differentiation in the skin^12^. Our results indicated that Ash1l, a known H3K27m3 antagonizer, is required for epidermal homeostasis. This gives more evidences on the other side of pendulum^30^.

Although we only showed impaired healing response in acute wound, the fact that skin lesions always originate from areas under chronic mechanical stimulations in aged *Ash1l* mutants suggests that chronic wound healing process is also disturbed. After wounding, *Ash1l*^*PB/PB*^ mice retained more proliferative cells in the epidermis (Fig. 5c–f). This result suggests that *Ash1l* is involved in regulating keratinocytes switching from proliferation to differentiation. Supporting this idea, we not only observed an inverse correlation between *Ash1l* expression and keratinocyte proliferation during wound healing (Fig. 5i), but also detected the specific effect of *Ash1l* mutation on c-Myc expression in lesion samples (Fig. 6a,b).

Keratinocyte overproliferation may reflect tumorigenesis, recruitment of quiescent stem cells into the cycle, or increased rounds of cell division. Striking similarities between wound healing and tumorigenesis have been reported in epidermis^31^. In addition, tumors can be developed at the site bearing chronic skin wounds^32^,^33^. However, tumorigenesis is unlikely involved in the case of *Ash1l* mutants, since the polarized expression of α6β4 integrin, a basement membrane protein that may expand suprabasally in malignant progression^34^, is not changed (Fig. S3). The LRC experiment excluded the possibility the *Ash1l*^*PB*/PB^ have extra amount of epidermal stem cells. Thus the thickened epidermis comprised more keratinocytes rather than neoplastic cells or activated stem cells.

It has been shown that the same *Ash1l* mutation may double IL-6 production of macrophages upon stimulation^35^. Considering that IL-6 could act to activate keratinocytes growth, it is possible that IL-6 may contribute to keratinocyte hyperproliferation and skin hyperplasia during skin wound healing. However, comparing with keratinocytes, macrophages in the dermis only act as a minor sources of IL-6^36^. In addition, IL-6 mediated responses in epidermis are largely compartmentalized^37^. Thus, the skin lesion presented in *Ash1l*^*PB*/PB^ mice is less likely caused by dysregulation of IL-6 in macrophages.

Our results prefer that c-Myc is an important downstream effector of *Ash1l* in regulating wound healing. As a methyltransferase, *Ash1l* can antagonize H3K27 methylation, a critical step for PcG dependent activation of *c-Myc,* by methylating H3K36. Consistently, we observed upregulation of H3K27 tri-methylation and c-Myc expression in *Ash1l* knock down cells. In addition, both *Ash1l* mutant and *c-Myc* transgenic mice showed increased cell proliferation with delayed cell differentiation and re-epithelialization in epidermis (Fig. 5i and ref. 6). *Ash1l* mutations do not lead to depleted stem cells, a phenomenon which has been observed after specific overexpression of MYC in the basal layer. This may suggest a dispensable role of *Ash1l* in stem cell maintenance.

The relation between Ash1l mutation and human epidermal diseases remains unclear. However, there have been reports suggesting the contribution of dysregulated Ashl1 expression to cell proliferation in liver, thyroid, breast, and esophageal cancers^38^,^39^,^40^,^41^. Therefore, it would not be surprising to detect the involvement of Ash1l in epidermal homeostasis and diseases in human.

## Materials and Methods

### Mice

The *Ash1l* mutant were generated as described^42^. In brief, a piggyBac transposon carrying the *Act-RFP-polyA* cassette was coinjected into the fertilized oocyte with a helper plasmid expressing transposase to get single copy insertions on the FVB/NJ background. Transposase free progeny carrying the insertion in the 15^th^ intron of the *Ash1l* gene (chromosome 3,88,860,750 bp, Ensembl release 45) was then bred for the experiments described here. The *Act-RFP-polyA* cassette sits in the same direction with that of *Ash1l* transcription, such that the polyA signal is expected to interfere with the expression of *Ash1l*. Adult mice were group housed and kept on 12/12-hour light/dark cycles. The use of mice was conducted in accordance with the regulations and guidelines approved by the Institute of Developmental Biology and Molecular Medicine Institutional Animal Care and Use Committee.

### BrdU treatment

To label replicating cells, 100 mg/kg BrdU was injected intraperitoneally 2 hours before the mice were sacrificed. For label retention studies, postnatal day 3 animals were injected with 50 mg/kg BrdU in 12 hours intervals, for 3 consecutive days, before the mice were euthanized 90 days later. At least three mice of each genotype were examined at each time point.

### Immunostaining

Freshly prepared skin samples were embedded in OTC and frozen in liquid-nitrogen-cooled isopentane. Sections (7 μm) were then collected and stained with either hematoxylin and Eosin or Oil-Red O. Immunofluorescent and immunohistochemical staining of frozen sections was carried out following standard protocols. For immunohistochemical analysis, the signal was visualized in a DAB color developing solution and counterstained with hematoxylin when necessary. To detect BrdU-labelled cells, after permeabilisation and prior to incubation with the anti-BrdU antibody, sections were incubated for 20-30 minutes in 2 M HCl at 37 °C. The following antibodies were used: rabbit anti-human ASH1L (ab4477, Abcam, 1:50), keratin 1 (MK1, Covance, 1:1000), keratin 14 (MK14, Covance, 1:1000), Loricrin (Loricrin, Covance, 1:1000), Ki67 (Ki67, Novocastra, 1:1000), BrdU (ab6326, Abcam, 1:500) and CD49f (Integrin α6, BD Pharmingen, 1:200), goat anti-rabbit IgG-FITC (Sigma, 1:1000), and donkey anti-rat IgG-FITC (Sigma, 1:1000).

### Real-time RT-PCR

Total RNA extraction and cDNA preparation were performed according to the manufacturer’s instructions with samples from E10.5 embryos or the skin. Real-time PCR was performed with ABsolute QPCR SYBR Green Mixes (ABgene) on an Mx3000P Quantitative PCR System (Stratagene). The primers used for PCR were: Ash1l-CDF (5′-GCC AGT CTT CAA GCA CGC ATA G-3′), Ash1l-CDB (5′-GTT CCT CTC TCT GTT GGA CAT TGG-3′), Actin-F (5′-AAG GCC AAC CGT GAA AAG AT-3′), and Actin-R (5′-GTG GTA CGA CCA GAG GCA TAC-3′). The 2^−ΔΔCT^ method^43^ has been used for relative mRNA quantification and Actin expression was measured as the internal control. The results represented three separate experiments conducted in triplicate.

### Western blot

Proteins from mouse skins were extracted by radioimmune precipitation assay (RIPA) buffer containing 1 mM PMSF and 1 proteinase inhibitor (Roche). The proteins were separated by electrophoresis on 10% SDS polyacrylamide gels and immunoblotted following standard protocols. The antibodies used were goat anti-human ASH1L (Santa Cruz sc-104089, 1:200), mouse anti-GAPDH (KangChengBiotech KC-5G4, 1:10,000), goat anti-mouse IgG-HRP (Santa Cruz sc-2005, 1:2000), and goat anti-rabbit IgG-HRP (Santa Cruz sc-2004, 1:2000).

### Barrier staining

Embryos were collected at E16.5 and washed in PBS before 12-hr incubation at 37 °C in 1 mg/ml X-gal (5-bromo-4-chloroe-indolyl- b,D-galactopyranoside, pH4.5). Embryos were then rinsed in PBS and fixed in formalin and photographed. This assay relies on the ability of X-gal to penetrate embryonic skin prior to barrier formation where X-gal is cleaved by endogenous β-galactosidase activity in the skin^22^.

### Wound-healing analysis

Four 3-mm punch biopsies (Miltex) were generated in the middle of the back of 12-week-old *Ash1l*^*PB/PB*^ mice and wild-type littermates as described^6^. Wounds were excised and section for H&E staining or BrdU analysis.

### Statistics

For microscopic counts of immunostaining data, the counting was performed on ten fields on a single sample and at least 3 samples were used for each experiment. The detailed sample size was listed in figure legends.

For all grouped comparison, two-tail unpaired Student’s *t*-test was used and data were presented as mean ± SED in figures. and Microsoft Excel and Graphpad Prism 6 was used for plotting. The significance is indicated with the following categories: (1) *p < 0.05; (2) **p < 0.01; (3) ***p < 0.005.

### RNAi constructs

The shRNA oligos were designed and cloned into pLKO.1 cloning vector as described^44^. The shRNA targeting sequences used included ASH1L RNAi-1: CCT GCC AAA TAC CAT AAG AAA; and ASH1L RNAi-2: AAT CGT GAA AGG AAC TTT GTG.

### Luciferase Assay

A 2.8 kb fragment containing c-Myc promoter was cloned into a luciferase vector pGL420 [luc2-Puro] to form the reporter plasmid pMycP-Luc. HEK293T cells were plated into 24-well plates and transfected with RNAi constructs (0.9 ug) and pMycP-Luc (0.2 ug). Luciferase activity in the cell lysates was assayed with the Luciferase Assay Reagent. To account for the differences resulting from transfection efficiency, all wells were also co-transfected with pCX-LacZ. The β-galactosidase activity was determined in buffer containing ONPG. The relative luciferase activity was thus defined as luciferase activity/β-galactosidase activity.

## Additional Information

**How to cite this article:** Li, G. *et al*. The Histone Methyltransferase Ash1l is Required for Epidermal Homeostasis in Mice. *Sci. Rep.* **7**, 45401; doi: 10.1038/srep45401 (2017).

**Publisher's note:** Springer Nature remains neutral with regard to jurisdictional claims in published maps and institutional affiliations.

## Supplementary Material

Supplementary Information
