# Supplementary material for: The Histone Methyltransferase Ash1l is Required for Epidermal Homeostasis in Mice
Source: Sci Rep. 2017 Apr 4;7:45401. doi: 10.1038/srep45401 (PMC5379632; doi:10.1038/srep45401)

The Histone Methyltransferase Ash1l is Required for Epidermal Homeostasis in Mice

Gang Li[1,#,*], Zhisheng Ye[1,#], Cheng Shi[1**], Ling Sun[1], Min Han[1,2], Yuan Zhuang[1,3], Tian Xu[1,4], Shimin Zhao[1], Xiaohui Wu[1]

[1]State Key Laboratory of Genetic Engineering and National Center for International Research of Development and Disease, Institute of Developmental Biology and Molecular Medicine, Collaborative Innovation Center for Genetics and Development, School of Life Sciences, Fudan University, Shanghai 200433, China;

[2]Howard Hughes Medical Institute, Department of Molecular, Cellular, Developmental Biology, University of Colorado, Boulder, CO 80309, USA;

[3]Department of Immunology, Duke University Medical Center, Durham, NC 27710, USA;

[4]Howard Hughes Medical Institute, Department of Genetics, Yale University School of Medicine, New Haven, CT 06536, USA.

[#] These authors contribute equally to this work.

* Present address: Section of Hematology/Oncology, Departments of Medicine and Molecular and Cellular Biology, Baylor College of Medicine, Houston, TX 77030, USA

** Present address: Lewis-Sigler Institute for Integrative Genomics, Department of Molecular Biology, Princeton University, Princeton, New Jersey 08544, USA

To whom correspondence may be addressed: E-mail: xiaohui_wu@fudan.edu.cn

SUPPLEMENTARY FIGURE LEGENDS

Figure S1. Disruption of Ash1l did not affect embryonic epidermal development. Immunofluorescent staining of keratinocyte markers K14, K1, and Loricrin did not detect significant changes of keratinocyte differentiation and hair follicle structure in E15 (a) and E17 (c) mutant embryos. X-gal staining revealed similar positions of the impermeability front (dashed lines) in E16.5 Ash1l$^{PB/PB}$ and wild-type embryos, indicating that epidermal barrier pattern formation was not affected by the Ash1l mutation. Scale bar= 50 µm

Figure S2. Ash1l deficiency did not alter the number of label retaining cells. Immunofluorescent staining did not detect location (a) and quantity (b) changes of label retaining cells (LRCs) in Ash1l$^{PB/PB}$ mice.

Figure S3. Ash1l$^{PB/PB}$ mice have intact epidermal basement membrane. The location and polarity of alpha6 integrin (a6) in the epidermis of Ash1l$^{PB/PB}$ mice were comparable to those of the wild-type littermates.

Figure S1

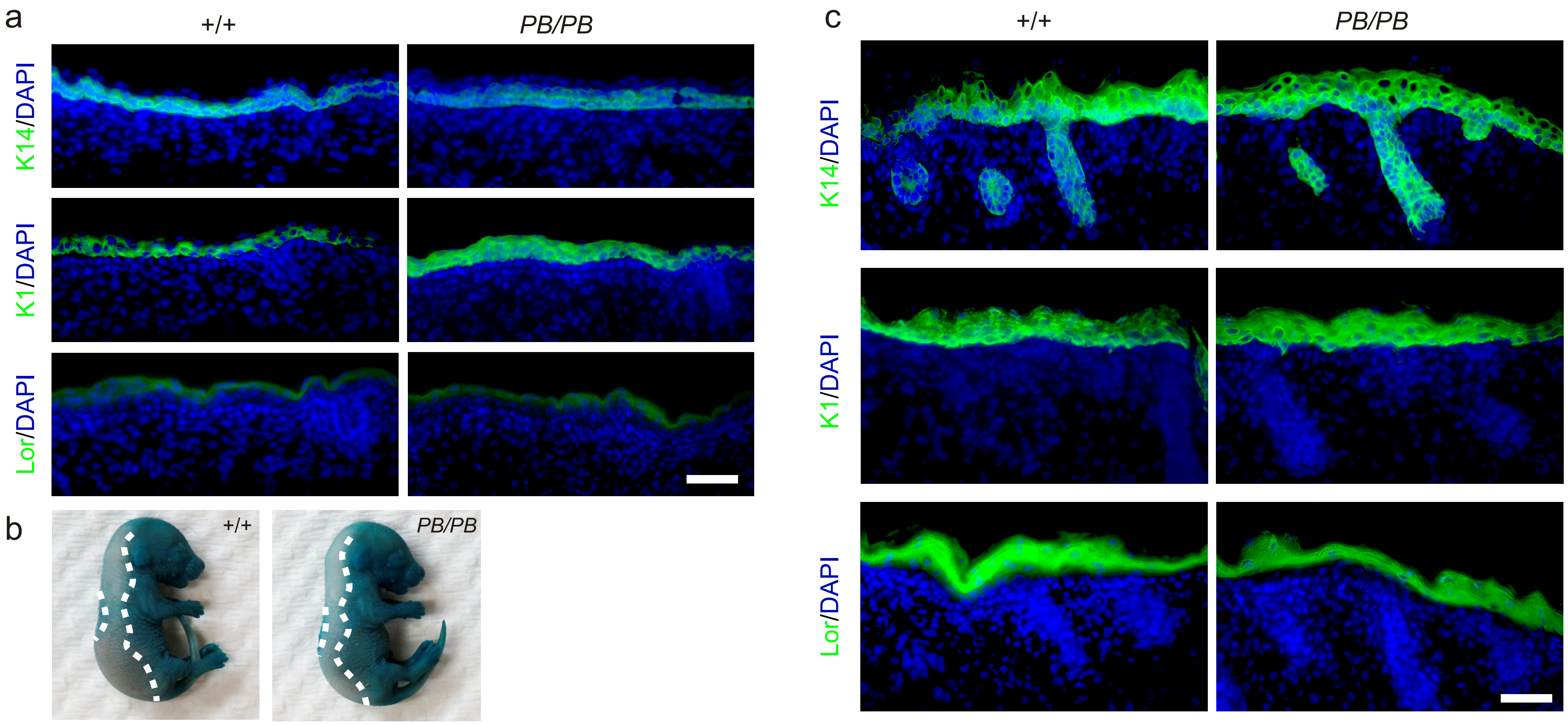
a
+/+
PB/PB
K14/DAPI
K1/DAPI
Lor/DAPI
b
+/+
PB/PB
c
+/+
PB/PB
K14/DAPI
K1/DAPI
Lor/DAPI

Figure S2

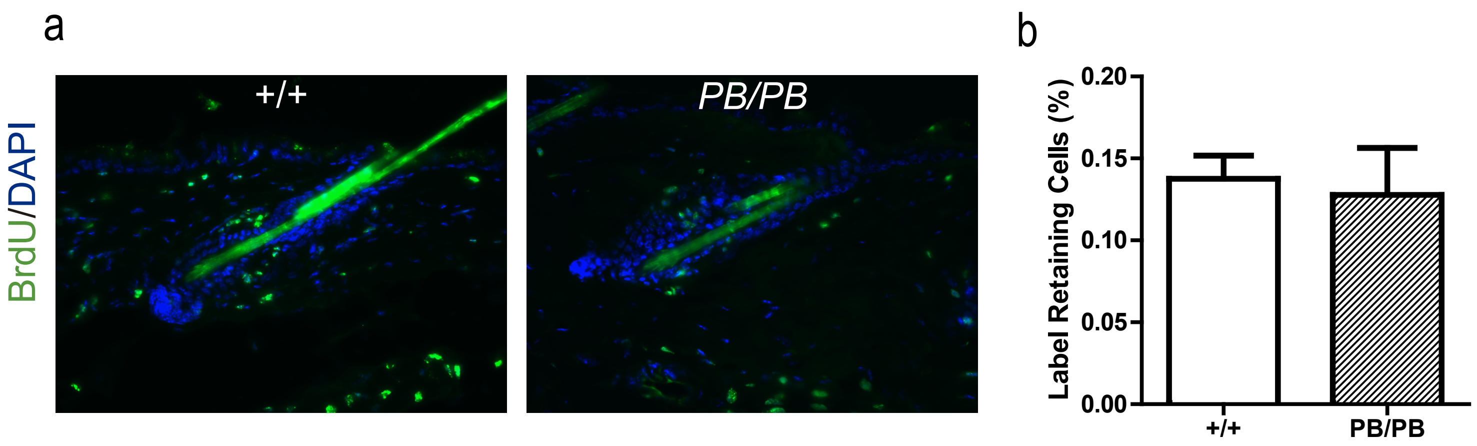
a
BrdU/DAPI
+/+
PB/PB
b
Label Retaining Cells (%)
0.20
0.15
0.10
0.05
0.00
+/+
PB/PB

Figure S3

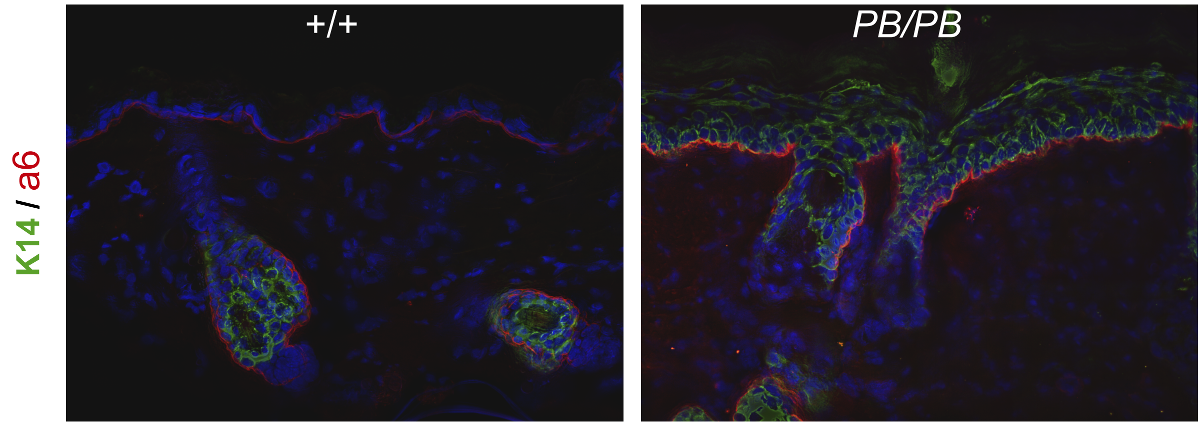

Supplement: Supplementary Information [file srep45401-s1.pdf]
